# A public dataset of dogs vital signs recorded with ultra wideband radar and reference sensors

**DOI:** 10.1038/s41597-024-02947-4

**Published:** 2024-01-22

**Authors:** Shahzad Ahmed, Seongkwon Yoon, Sung Ho Cho

**Affiliations:** Department of Electronic Engineering, Hanyang University, Seoul, 04763 South Korea

**Keywords:** Biomedical engineering, Rehabilitation

## Abstract

Recently, radar sensors have been extensively used for vital sign monitoring in dogs, owing to their noncontact and noninvasive nature. However, a public dataset on dog vital signs has yet to be proposed since capturing data from dogs requires special training and approval. This work presents the first ever ultra wideband radar-based dog vital sign (UWB-DVS) dataset, which was captured in two independent scenarios. In the first scenario, clinical reference sensors are attached to the fainted dogs, and data from UWB radar and reference sensors are captured synchronously. In the second scenario, the dogs can move freely, and video recordings are provided as a reference for movement detection and breathing extraction. For technical validation, a high correlation, above 0.9, is found between the radar and clinical reference sensors for both the heart rate and breathing rate measurements in scenario 1. In scenario 2, the vital signs and movement of the dogs are shown in the form of dashboards, demonstrating the long-term monitoring capability of the radar sensor.

## Background & Summary

Adoption of pets in the high-income countries has become so prevalent that pets are considered to be the family members and given special care^1^. In particular, dogs have a special attachment to humans, and due to their friendly and supportive nature, they are termed “man’s best friend”. The prior literature supports the rationale that interaction with a therapy dog significantly reduces the level of stress among professionals with difficult jobs^2^. Owing to the multi-fold benefits of petting a dog, statistics indicate that the number of pet dogs is increasing in developed countries, which is raising concerns related to the health and well-being of dog^3^. Unlike humans, dogs cannot express their health status easily and therefore require special care. Vital signs (VS), which include the breathing rate (BR) and heart rate (HR), are amongst the key markers of a dog’s well-being^4^. Research indicates that a substantial rise or fall in HR or BR can be used to assess the health condition and emotional state of dogs^5,6^.

Among the available VS monitoring sensors, radar offers a noncontact and noninvasive solution. Among different radar technologies, impulse radio ultrawideband (IR-UWB) radar is tolerant of ambient noise and multipath effects, making it well suited for home use. In the literature, IR-UWB radar has shown its usefulness in several short-range applications, such as gesture recognition^7,8^, fall detection^9^ and VS measurement^10^.

The history of measuring dogs’ vital signs (DVS) using radar dates to 1975 when Lin^11^ extracted the breathing rate of dogs using microwaves. At present, a considerable amount of work has been performed for radar-based non-contact and non-invasive VS measurement of DVS^1,12,13^. For example, Wang *et al*.^5^ demonstrated VS monitoring of dogs and cats using UWB radar. Similarly, several other researchers have also considered DVS monitoring using radar^1,13^.

Currently, several open-source datasets of radar-based human VS exist in the literature. A dataset of heart movement recorded with radar along with a synchronized references sensor was proposed by Shi *et al*.^14^. Another dataset with clinical verification for human VS was proposed by Schellenberger and coworkers^15^. In addition to that, children VS dataset also exists^16^. While a few radar-based human VS datasets exist in the literature, radar-based dogs VS and related activity datasets are still lacking. VS extraction for human and dogs are currently being studied separately due to several differences. Consequently, a dedicated dogs VS dataset is also required.

**Fig. 1:**
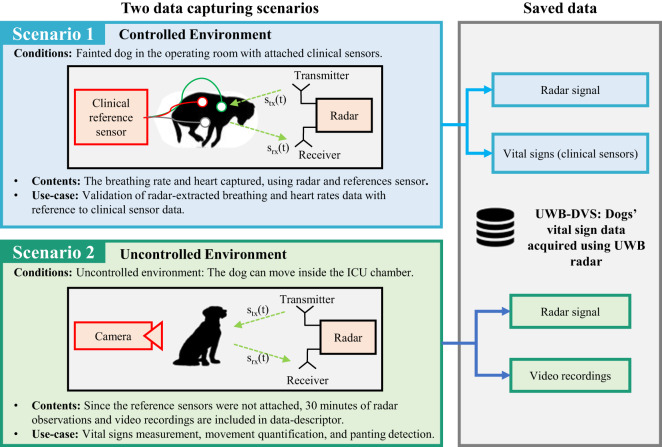
Summary of two data capturing setups (fainted dogs and awake dogs).

UWB-DVS provides the first ever VS dataset of dogs acquired using UWB radar and reference sensors simultaneously. The off-the-shelf (UWB) radar sensor opted in this study comprises of nearly “all-digital” components and have minimal radio frequency components, making the circuitry simple. Moreover, UWB radars can function effectively in environments with radio frequency (RF) noise, such as homes and offices. To provide rich information related to dogs’ vital signs and behavior, two scenarios are created as shown in Fig. 1. The details of each scenario are as follows

- Scenario 1 (Controlled environment with fainted dogs): Data from fainted dogs in the operating room are collected using radar and clinical reference sensors as shown in Fig. 1. Existing studies often consider VS extraction from fainted dogs^5^. In this work, general anesthesia along with Propofol as an additional induction agent, was used to induce unconsciousness in the dogs.
- Scenario 2 (Uncontrolled environment with awake dogs): The dog is kept unconstrained inside the Intensive Care Unit (ICU) chamber and data are captured for 30 minutes. Since the dog is awake and conscious, Electrocardiogram (ECG) is not attached in this scenario; instead, a camera is attached to observe VS and movement. The second scenario can provide a continuous personalized VS measurement method for a dogs. To show the continuous monitoring solution, we also propose a dashboard for scenario 2, showing the radar observed dog’s movements and VS measurements.

## Methods

### Use-cases of both scenarios

Both scenarios have individual importance for DVS monitoring applications. The data corresponding to Scenario 1 can be used to perform preclinical validation of DVS extraction algorithms. It is common practice in literature to benchmark the algorithms on the fainted dogs in the clinical environment^5^. The radar and reference sensors are synchronized in time so that the error and correlation between the two sensors can be computed.

On the other hand, scenario 2 can be considered as a daily monitoring use-case scenario. Long-term observations can be used to monitor movements, frequency of restlessness and VS for a prolonged period of time. Although the clinical reference data are not available in scenario 2 since clinical sensors worn on the bodies of moving dogs are not recommended by medical experts, the captured data can be used to determine the personalized baseline of each dog, and sudden change in VS or movement history can be used as an early indicator of a hazardous situation. In addition to that, camera sensor can be used to observe the movements being made by the dog. To evaluate the accuracy of a specific VS extraction algorithm, scenario 1 must be considered.

### Data Acquisition Environments and Hardware

#### Data acquisition environments

**Fig. 2:**
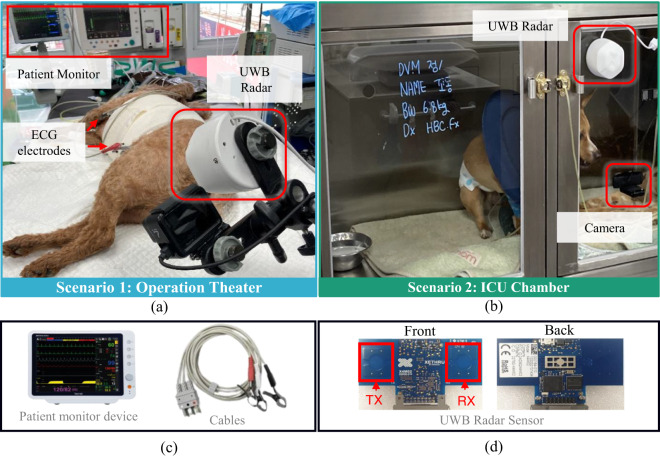
Data capturing environments and equipment: (**a**) Fainted dog in an operating room, (**b**) dog placed inside the hospital care unit (uncontrolled environment), (**c**) reference sensors for (**a**), and (**d**) IR-UWB radar used in (**a**) and (**b**).

The data acquisition environments and the apparatus are shown in Fig. 2. For scenario 1, shown in Fig. 2a, the data are collected in the operating room and the reference sensors were connected to the fainted dog using ECG electrodes. The radar sensor and camera are installed at a distance of 0.3 meters as shown in Fig. 2. The second scenario is shown in Fig. 2b, where the dog is awake and can make movements (freely) inside the ICU chamber. Since the installation of body worn sensor is not feasible when the dog is awake, a camera sensor is attached as a reference which can show the movement of the dog and the movement of the lungs when the dog is at rest.

#### Reference sensors

For scenario 1, a clinical reference sensor for HR and BR extraction was attached to the body of each dog as shown in Fig. 2a. We used a BM7Vet Pro ECG sensor, manufactured by Bionet Co., Ltd, shown in Fig. 2c. The device is a clinically approved veterinary VS monitoring sensor, which is extensively used at the health care facilities throughout the world.

For scenario 2, video recordings for each dog are included in the UWB-DVS data descriptor. Veterinarians often measure the BR and HR of dogs by manually observing the pulse for 10 seconds and multiply the result by six. From video recordings, one may approximate the BR in a similar way. The video recordings additionally act as ground-truth values for the dogs’ movement history quantification.

#### Radar sensor

In this study, we used a Xethru-X4 IR-UWB radar device designed by Novelda, Norway, consisting of a single transmitter and receiver pair as shown in Fig. 2d. Xethru radar has been extensively used in the literature for several applications including human VS measurement^17^, dog VS measurement^5,18^ and gesture recognition^7,19^. The radar operates at a center frequency of 7.29 GHz. The rest of the settings are summarized in Table 1.

**Table 1:** IR-UWB radar parameter settings for capturing the UWB-DVS data descriptor.

Parameter	Value
Frame rate	50 frames/second
Center frequency	8.75 GHz
Sampling frequency	23.32 GHz
Bandwidth	1.50 GHz (−10 dB)
Pulse repetition interval	40.50 MHz
Transmitter-receiver pair	Single pair

#### Radar and reference sensor synchronization

The clocks of radar and reference sensors were synchronized up to fractions of seconds. The medical device operator and the radar engineer synchronized and verified the timestamps. The radar data were sampled at 50 FPS (see Table 1), whereas the reference data were sampled at 1 FPS. Prior to statistically validating the data, the radar extracted vital signs were averaged to 1 FPS in order to match the reference sensor data.

### Radar Signal Modeling

The dog’s skin vibrates continuously because of heart and lungs movement. This vibration *x*(*t*) can be defined as^20^

1 $$x(t)={x}_{o}+{A}_{br}{\rm{s}}{\rm{i}}{\rm{n}}(2\pi {f}_{br}t)+{A}_{hr}{\rm{s}}{\rm{i}}{\rm{n}}(2\pi {f}_{hr}t),$$

where *x*_*o*_ represents the mean value of distance around which the body of dog vibrates. The terms *A*_*br*_ and *A*_*hr*_ represent the amplitudes of the signal generated by the movements of the lungs and heart respectively. The corresponding movement frequencies for the heart and lungs are represented as *f*_*br*_ and *f*_*hr*_ respectively. With the dog as the only moving object in the radar cross-section (RCS) as shown in Fig. 2, the impulse response of the system will be

2 $$h(\tau ,t)={a}_{vs}\delta (\tau -{\tau }_{vs}(t))+clutter.$$

Here, the impulse response corresponding to the dog vital signs is termed as ${a}_{vs}\delta (\tau -{\tau }_{vs}(t))$ and the clutter will is

3 $$clutter=\sum _{i}{a}_{i}\delta (\tau -{t}_{i}),$$

where *a*_*i*_ and *t*_*i*_ respectively represent the amplitude and time of the *i*_*th*_ reflection. With *c* being the speed of light, the time delay between the transmitted and received signals *τ*_*vs*_ is represented as:

4 $${\tau }_{vs}=\frac{2x(t)}{c}={\tau }_{o}+{\tau }_{br}sin(2\pi {f}_{br}t)+{\tau }_{hr}sin(2\pi {f}_{hr})$$

where *τ*_*br*_ and *τ*_*hr*_ represent the time delays due to the BR and HR, respectively. The signal at the receiver of radar, represented in Fig. 1 as *s*_*rx*_(*t*), will be the result of convolution of the transmitted signal *S*_*tr*_(*t*) and the system impulse response *h*(*τ*,*t*)

5 $${s}_{rx}(\tau ,t)={s}_{tx}(\tau )\ast h(\tau ,t)+clutter.$$

6 $${s}_{rx}(\tau ,t)={a}_{vs}{s}_{tx}(\tau -{\tau }_{vs}(t))+clutter.$$

Here, *S*_*tx*_(*τ*) is the shape of the transmitted signal. Equation 6 shows a single pulse being transmitted by radar. Several pulses are often transmitted, and the received response is stacked as a two dimensional (2D) matrix which (in discrete form) can be expressed as

7 $$R[n,m]={S}_{rx}[m,n]+c[m],$$

where *R*[*n*,*m*] is a 2D matrix with *n* rows and *m* columns, termed as slow-time and fast-time respectively. Note that each row of Eq. 7 contains the vital sign reflections for one transmitted frame as expressed in 6. These frames are repeated continuously and in our case, 50 frames per seconds are being saved, as reported in Table 1.

### Dogs Vital Sign Extraction

**Fig. 3:**
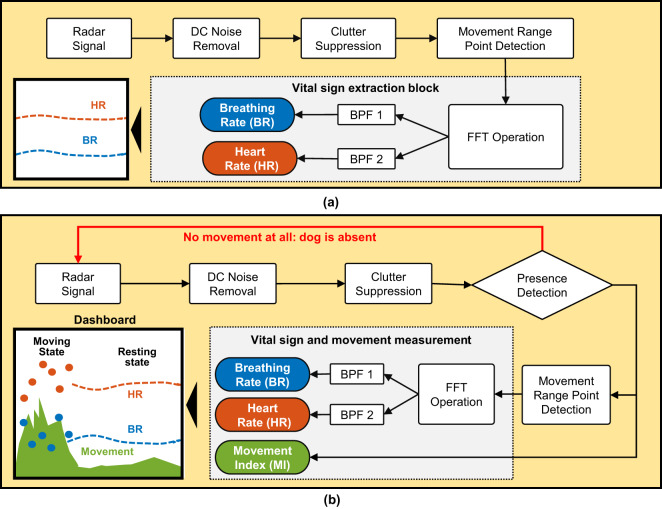
Simultaneous extraction of heart rate and breathing rate using IR-UWB radar for (**a**) fainted dogs and (**b**) awake dogs.

The vital sign extraction algorithms for scenarios 1 and 2 are illustrated in Fig. 3a and b, respectively. The 2D matrix shown in Eq. 7 serves as the input to the algorithm in both scenarios.

For the static case, we removed the DC component of the signal prior to determining the movement made in front of the radar. Afterward, the clutter term *c*[*m*] consisting of static reflections was suppressed by deploying a loop-back recursive filter^7,16^.The target (dog’s) position was determined by calculating the variance of the 2D radar data matrix *R*[*n*,*m*], and the distance showing the highest variance was selected as the target range bin.

After determining the location of the dog, the fast Fourier transform (FFT) was used to quantify the frequency of body vibrations. The BR and HR of a normal dog range between 10–40 Hertz (Hz) and 60–160 Hz respectively. Consequently, two bandpass filters (BPF) were applied in the frequency domain to separate the BR and HR. The whole operation was repeated over a sliding window for continuous monitoring purposes.

The vital signs extraction procedure for scenario 2 is also based on the similar concept opted in scenario 1 however, prior to vital sign extraction, the movement was quantified and plotted along with the vital signs as illustrated in Fig. 3b. The movement of dog under test was determined by taking the absolute sum of the difference of two consecutive frames^21^

8 $$MI(n)=| \sum {R}_{cr}[n,m]-{R}_{cr}[n-1,m]| ,$$

where *R*_*cr*_ is the clutter-removed version of radar data matrix *R* described in Eq. 7, and the amplitude of *MI* quantifies the movements of the dog. As stated earlier, the terms *n* and *m* represent the fast-time and slow-time respectively. The magnitude of *MI* defines whether the dog is absent, moving or resting. As shown in Fig. 3, stable vital signs can be observed only when the dog is resting. The dashboard shows the insight about the movement and dogs’ VS concurrently. For instance, any abrupt change or anomaly in the dog’s VS when the movement index is considerably low may be an early indicator of a possible medical emergency.

### Data acquisition procedure and protocols

Data collection was performed at the Nowon N animal hospital, Seoul, South Korea. All the procedures and protocols were approved by the Hanyang University Institutional Animal Care and Use Committee (HYU-IACUC;2022-0252), and the researchers were strictly advised to follow the established guidelines throughout the process. Two medical experts and a radar engineer supervised the data capturing process, and the data were selected by their mutual approval. Overall data acquisition was supervised by the corresponding author. As stated earlier, we captured data in two different environments, and the procedure and protocol for each environment are discussed individually in subsections for convenience.

#### Scenario 1: Constrained environment

In this scenario, data from (anaesthetized) fainted dogs were captured and the reference sensors were attached to the dog’s body. In the literature, data from fainted dogs are often collected to quantify the performance of VS extraction algorithms.

The dogs were brought to the operating room and received anaesthesia followed by the surgery (See Table 2). Afterwards, the fainted dogs were placed in front of the radar sensor for three minutes. The captured reference sensor and radar data were subjected to quality control. If either of sensor’s data was missing or invalid, the data collection process was repeated again.

**Table 2:** Details of the participants involved in scenario 1.

Dog no.	Age (years)	Breed	Gender	Weight (Kg)	Medical Condition
1	7	Dachshund	Male	11.3	Disc surgery
2	4	Shiba dog	Female	12.0	Chylothorax treatment
3	8	Poodle	Female	8.7	MGT(Mammary Gland Tumor)
4	5	Poodle	Female	2.9	Left Cruciate Ligament Rupture
5	8	Poodle	Female	8.3	Cholecystectomy surgery
6	4	Schnauzer	Female	5.5	Neutering
7	7	Maltese	Female	3.8	Cruciate Ligament Rupture
8	12	Pomeranian	Female	3.2	Canine Pyometra
9	8	Poodle	Female	6.0	Tooth Extraction, Neutering
10	4	Pompitz	Female	5.1	Right Cruciate Ligament Rupture

#### Scenario 2: Unconstrained environment

As shown in Fig. 1, the dogs in this scenario were kept awake and they could move freely within an enclosed (ICU) chamber. Body-worn sensors were not attached; instead, a camera sensor was installed as a reference sensor. Medical experts quantified the breathing rate of the dog by observing the video frames. Note that before capturing data, the dogs underwent the surgery first and were brought to the ICU chamber where radar and a camera sensor was installed to capture data.

For performance benchmarking of HR and BR, the data collected in scenario 1 must be considered. However, the radar data collected in scenario 2 can be used to extract BR and HR while the ground truth values are available only for the BR.

### Participants detail

Tables 2 and 3 shows the involved dogs for scenario 1 and 2 respectively. Ten dogs were included in scenario 1 and twenty dogs were included in scenario 2. Data collection was performed in the hospital on walk-in dogs, and their medical conditions are also included in Table 2. Note that all ten dogs in scenario 1 were in fainted condition. All the dogs employed in this work were small or medium to small sized dogs.

**Table 3:** Details of the participants involved in scenario 2.

Dog no.	Age (years)	Breed	Gender	Weight (Kg)	Medical Condition
11	9	Shih Tzu	Female	7.4	Hospitalization for surgery
12	4	Maltese	Male	10.7	Hospitalization for surgery
13	10	Mixed Breed	Female	8.2	Hospitalization for surgery
14	10	Pomeranian	Female	8.1	Hospitalization for surgery
15	12	Mixed Breed	Female	11.3	Hospitalization for surgery
16	8	Maltese	Male	7.6	Hospitalization for surgery
17	5	Mixed Breed	Male	16.1	Hospitalization for surgery
18	7	Mixed Breed	Female	7.7	Hospitalization for surgery
19	6	Hound	Female	12.2	Hospitalization for surgery
20	10	Maltese	Female	9.9	Hospitalization for surgery
21	9	Maltese	Female	7.2	Hospitalization for surgery
22	10	Maltese	Female	13.7	Hospitalization for surgery
23	4	Shiva Dog	Female	12.1	Hospitalization for surgery
24	5	Maltese	Male	13.2	Hospitalization for surgery
25	5	Welsh Corgi	Female	8.5	Hospitalization for surgery
26	11	Poodle	Male	11.5	Hospitalization for surgery
27	10	Maltese	Male	7.6	Hospitalization for surgery
28	6	Dachshund	Male	13.5	Hospitalization for surgery
29	8	Pomeranian	Male	7.4	Hospitalization for surgery
30	7	Maltese	Female	6.6	Hospitalization for surgery

## Data Records

**Fig. 4:**
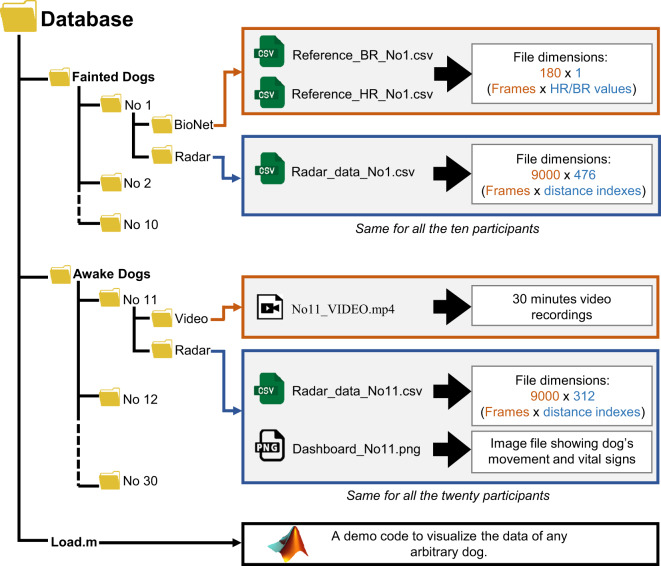
The structure of the UWB-DVS dataset.

The dataset is publicly available for download at the figshare repository^22^. The structure of the public repository is shown in Fig. 4. Two folders are created for each scenario shown in Fig. 1, and all the associated data are placed inside each folder. The Bionet folder contains the reference sensors data, and the folder named ‘Radar’ contains the radar data. Since three minutes of data were captured for scenario 1, the reference sensor data includes 180 samples. On the other hand, the radar data file consists of 9000 samples. For scenario 2, the data are recorded for thirty minutes, resulting in 90,000 radar samples, as illustrated in Fig. 4.

## Technical Validation

### Validation of the data collected in scenario 1

The simultaneously collected data from radar and clinical sensors are first synchronized in time, allowing us to compute the mean absolute error (MAE) and interclass correlation (ICCR). These two factors are extensively used in the literature to measure the similarity of two measurements^14,16^.

Table 4 shows the average MAE for BR and HR. The MAE between the radar and reference sensor for BR is 2.3 breaths per minute whereas for heart rate, the MAE is approximately 3.7 beats per minute. Participants two, eight, and nine showed the highest error in BR. For HR, participant five had the highest error of 6.6 beats per minute. The overall error for both the HR and BR is within the tolerable range.

**Table 4:** Mean absolute error for heart rate and breathing rate measured in controlled environment (fainted dogs scenario).

Participant	MAE BR	MAE HR
Participant 1	1.8	1.2
Participant 2	3.4	1.7
Participant 3	2.2	4.6
Participant 4	1.8	3.0
Participant 5	2.4	6.6
Participant 6	2.6	5.8
Participant 7	0.6	5.0
Participant 8	3.3	2.3
Participant 9	3.5	4.4
Participant 10	1.5	2.5
Average	2.31	3.70

MAE is an extensively used parameter for determining the limits of agreement between radar and reference sensor values for VS measurement. However, the MAE cannot quantify the ability of radar to track changes in VS measurements. Consequently, we also consider scatter plots, Bland–Altman plots, and the corresponding ICCR values in addition to the MAE for the fainted dog scenario.

**Fig. 5:**
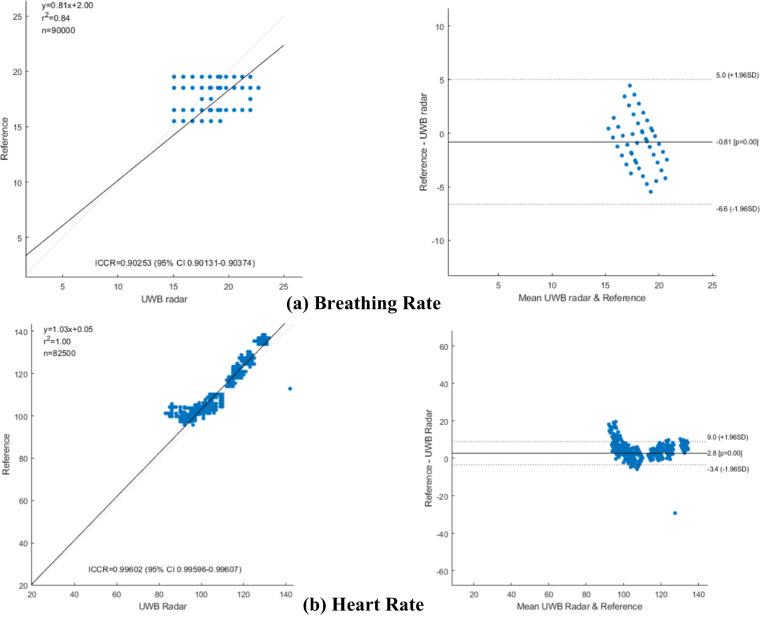
Scatter and Bland-Altman plots for (**a**) breathing rate and (**b**) heart rate data captured in scenario 1.

Figure 5a,b shows the scatter plots and Bland–Altman plots for HR and BR, respectively. In the scatter plots, the vertical axis shows the clinical reference sensor observations, and the horizontal axis shows the radar observations. The thick black line shows the ideal case where the two quantities have a 100 percent match. In the Bland-Altman plots shown in Fig. 5a,b, the vertical axis represents the difference in the VS observations between the radar and reference sensor. The horizontal axis represents the absolute values of VS observations. In the ideal case, the difference should be zero, and the deviation from the ideal values must be within a tolerable range. As shown in Fig. 5, the BR and HR of our sample space spans between 12 and 24 breaths per minute and 100 and 150 beats per minute respectively.

The ICCR coefficients shown in Fig. 5a for the BR and HR is 0.902 and 0.930 respectively, and the dotted curves are near to the strong black line, suggesting a high correlation coefficient. Similarly, the deviation shown in the Bland-Altman plots is also less than 5 beats per minute. It can be concluded that the radar data in this data descriptor can be used for benchmarking the VS extraction algorithms.

Reliable measurement is an important aspect of any medical device. The Association for the Advancement of Medical Instrumentation (AAMI) and American National Standards Institute (ANSI) suggest that the error in terms of percentage for a consumer device must be < ± 10% for human HR measurements^23^. The average MAE for BR and HR in our case was 2.3 and 3.7, respectively. Perhaps apart from one or two cases that had higher error, the error was always low, and lies within the tolerance range. For the ICCR, any value above 0.7 is considered to indicate a high correlation between the two devices under observation. In our case, the ICCR value was above 0.9, suggesting a high correlation between the two measurements.

### Validation of data collected in scenario 2

**Fig. 6:**
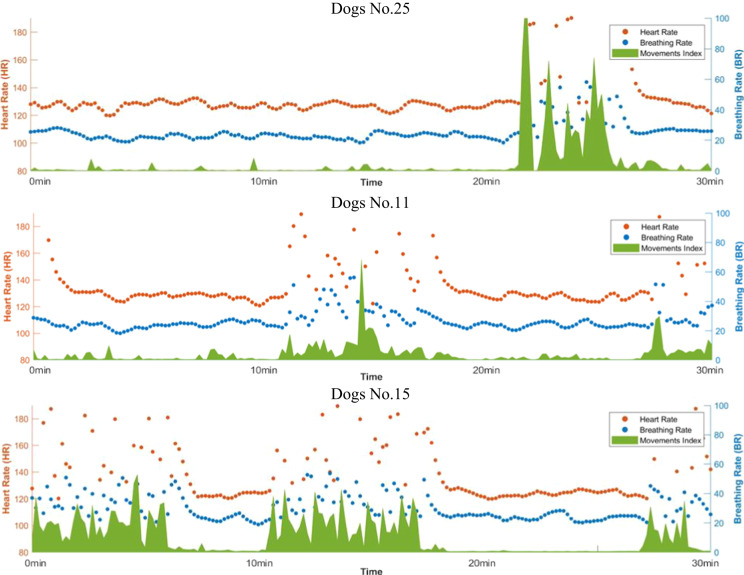
Dashboards showing the vital signs and movement history of dogs twenty five, eleven, and fifteen.

Figure 6 shows a thirty-minute history of three dogs chosen on the basis of the amount of movement made during their thirty minutes of stay in the ICU chamber shown in Fig. 2. Here, the green curve shows the body movement while staying inside the ICU chamber. The blue and brown dots show the HR and BR respectively. The HR and BR data points represent the average observations for ten seconds. As shown in Fig. 6, dog number 25 shows a very small amount of movement after 22 minutes of stay in the ICU chamber. A stable HR and BR are observed when the *MI* is low, which is the case when the dog is resting. Similarly, the dashboard for dog number 11 shown in Fig. 6 shows two instances when the dog is moving. Finally, the dashboard for dog number 15 shows several sharp movements with high magnitude. During these movements, the VS data appear like noise.

The dashboards shown in Fig. 6 suggest that radar sensors can be used for long term monitoring purposes. VS and body movement can be monitored in a continuous fashion, and the constructed dashboard can reflect the health status of dogs. Any abrupt changes in the VS or movement history can be observed on these dashboards and can be used as early indicators of hazardous situations. For instance, it was observed that dog number 15 made several movements while staying inside the ICU chamber, which can indicate that the dog is in a state of restlessness. Although we keep measuring vital signs when the dog is moving, the VS must be considered valid only when the movement index is also low, as illustrated in Fig. 6.

## Usage Notes

We recommend the use of scenario 1 for benchmarking and validation purposes, followed by real-world implementation on scenario 2. The data in the second scenario are not for clinical validation of the algorithms since the data are captured without (clinical) reference sensor. Instead, scenario 2 can be used to test the feasibility of long-term monitoring using radar sensors. As shown earlier in Fig. 6, the vital signs and movement index extracted in scenario 2 are used to observe how stable the dog is.

The first step in processing the received data is the removal of clutter. The clutter removal filter is a simple recursive loop-back filter that estimates the clutter based on the current and previous output^24^. For convenience, the data are saved in an open-source CSV file format. We used the MATLAB 2021b software package for processing the data, and the script to load the data is included in the data descriptor. However, any suitable software package can be considered for this purpose.

## Data Availability

A MATLAB file named as load.m is included in the repository, as shown in Fig. 4. After extracting the dataset on the local computer, the users must run the load.m file using MATLAB R2020 or later. The file will automatically load the entire dataset into the MATLAB workspace.
