# Author Correction: A public dataset of dogs vital signs recorded with ultra wideband radar and reference sensors

**DOI:** 10.1038/s41597-024-02993-y

**Published:** 2024-01-29

**Authors:** Shahzad Ahmed, Seongkwon Yoon, Sung Ho Cho

**Affiliations:** Department of Electronic Engineering, Hanyang University, Seoul, 04763 South Korea

**Keywords:** Biomedical engineering, Rehabilitation, Biomedical engineering, Rehabilitation

Correction to: *Scientific Data* 10.1038/s41597-024-02947-4, published online 22 January 2024

In the original version of the paper, the e-mail addresses of all authors were incorrectly included, rather than just Professor Sung Ho Cho as the corresponding author. This has been updated in the HTML and PDF versions of the paper.

